# Realization of palladium-based optomechanical cantilever hydrogen sensor

**DOI:** 10.1038/micronano.2016.87

**Published:** 2017-03-27

**Authors:** Steven J. McKeown, Xiaozhen Wang, Xin Yu, Lynford L. Goddard

**Affiliations:** 1 Photonic Systems Laboratory, Department of Electrical and Computer Engineering, Micro and Nanotechnology Lab, University of Illinois at Urbana-Champaign, 208 North Wright Street, MNTL 2254, Urbana, IL 61801, USA

**Keywords:** hydrogen detection, imaging and sensing, interference microscopy, optomechanics, optical sensors, surface dynamics

## Abstract

Hydrogen has attracted attention as an alternative fuel source and as an energy storage medium. However, the flammability of hydrogen at low concentrations makes it a safety concern. Thus, gas concentration measurements are a vital safety issue. Here we present the experimental realization of a palladium thin film cantilever optomechanical hydrogen gas sensor. We measured the instantaneous shape of the cantilever to nanometer-level accuracy using diffraction phase microscopy. Thus, we were able to quantify changes in the curvature of the cantilever as a function of hydrogen concentration and observed that the sensor’s minimum detection limit was well below the 250 p.p.m. limit of our test equipment. Using the change in curvature versus the hydrogen curve for calibration, we accurately determined the hydrogen concentrations for a random sequence of exposures. In addition, we calculated the change in film stress as a function of hydrogen concentration and observed a greater sensitivity at lower concentrations.

## Introduction

Hydrogen has always been viewed as a promising alternative to fossil fuels, and it can also function as an effective energy storage medium for intermittent energy sources. In addition, hydrogen is used in a range of other industries, including chemical production, metal refining, and food processing. A major safety concern with hydrogen is combustibility. Therefore, early leak detection and concentration determination of hydrogen have been areas of intense research^1–3^. There are various types of hydrogen sensors that use a wide range of detection mechanisms. Lundström *et al*.^4^ proposed metal oxide semiconductor (MOS)-type hydrogen sensors^5^. However, MOS sensors suffer from drawbacks such as premature saturation of detectable hydrogen concentrations and low sensitivity. Other MOS-based devices have been used as hydrogen sensors, such as MOS field-effect transistors (FETs)^6,7^, high electron mobility transistors^8–10^, and Schottky diode-type FETs^11,12^. However, these devices require complicated fabrication processes and have high production costs. Optical gas sensors^13–21^ not only overcome these disadvantages but also have other unique advantages, such as negligible electrical interference, no risk of ignition from an electrical spark, and the ability to work at high temperatures or in harsh environments.

Palladium (Pd) can absorb up to 900 times its own weight in hydrogen gas at room temperature^22^. Compared with platinum^23^, Pd film is more popular because of its lower cost. Pd alloys with Ag, Au, Ni, and WO_3_ have also been studied^24–29^ because of their improved response time^24,25,27^, sensitivity^28,29^, and durability^26^ as a sensing material. The alloys can avoid blistering effects^26^ and the *α* to *β* phase transition at higher hydrogen concentrations^25,27^. During the adsorption process, Pd hydride (PdH) is formed and the optical and mechanical properties of the Pd film are changed^24,30^. PdH can exist in the pure *α* phase, the mixed *α*+*β* phase, or the pure *β* phase when the ambient hydrogen concentration is low, medium, or high, respectively. The phase transition points can occur anywhere from 0.1 to 2% hydrogen depending on the thickness, quality, and structure of the film^30–32^. When the concentration is below the transition point for the pure *β* phase, the optical and mechanical properties of the Pd film will recover after hydrogen removal^13,14^. This property makes a Pd film an effective functional layer for optical hydrogen detection^3,31,33^. Pd-coated cantilevers were previously studied as capacitive hydrogen sensors^34–36^. In addition to changes in reflectivity, stress induced by hydrogen-induced lattice expansion (HILE) causes the cantilever to deform. Our group previously measured HILE in Pd microdiscs^37^.

Here we fabricated nanoscale Pd-coated cantilevers and characterized the devices using diffraction phase microscopy (DPM)^37–43^. The DPM technique achieves real-time single-shot non-destructive quantitative phase imaging *in situ* with nanometer-level sensitivity. The novelty of this work is the sensing modality because we can measure the instantaneous three-dimensional shape of the cantilever and use it to determine the change in curvature of the Pd film as a function of H_2_ concentration. Furthermore, we successfully demonstrated that random hydrogen concentrations can be accurately determined using the measured curvature changes of the Pd film cantilever. In addition, we used the curvature data to extract the change in residual stress of the Pd film as a function of hydrogen concentration.

## Materials and methods

**Figure 1:**
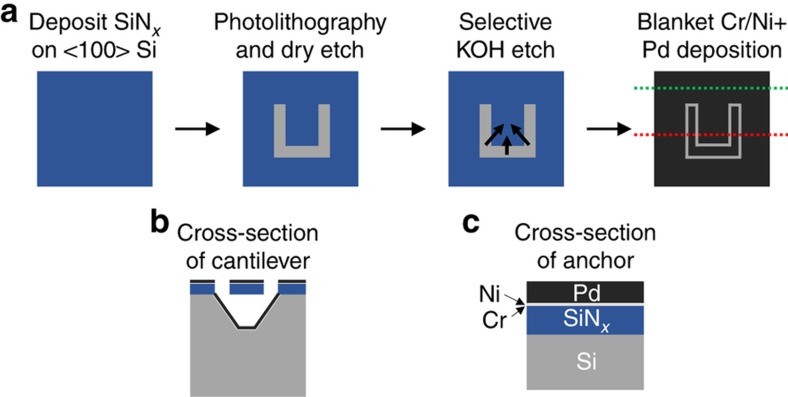
(**a**) Fabrication process flow. Cross-section of (**b**) the cantilever and (**c**) the anchor, through the red and green dotted lines, respectively, in the last image of (**a**). Pd, palladium.

Several cantilever geometries were simulated based on previous measurements^37^ on the expansion of Pd films. The important physical parameters are the cantilever’s beam material, film thicknesses, and length^44^. A process flow for the fabrication can be seen in Figure 1. Cantilevers were fabricated on 〈100〉 silicon using an anisotropic KOH etch process, with the 〈111〉 plane forming the sidewalls of the cavity under the cantilever. First, the silicon nitride (SiN*_x_*) cantilever layer was deposited via plasma-enhanced chemical vapor deposition (PECVD) using a Mesc Multiplex PECVD tool (Surface Technology Systems (STS), Stratham, NH, USA). After calibrating the deposition rate on a dummy sample, the deposition time was computed and used to obtain the desired nitride thickness on the real sample. Silicon nitride was selected as the beam material because of its compatibility with the KOH-based fabrication process and because the intrinsic stress in the film can be minimized by tuning the deposition conditions. Specifically, we used mixed RF frequencies of 13.56 and 380 kHz to minimize the intrinsic film stress. At this point, the intrinsic film stress was measured to be +190 MPa using a 500TC stress measurement tool (Frontier Semiconductor Inc., San Jose, CA, USA); the thickness was confirmed to be 1010 nm using an LSE-USB ellipsometer (Gaertner Scientific Corporation, Skokie, IL, USA). Next, contact photolithography was used to define C-shaped etch windows that were etched through the SiN*_x_* layer using reactive ion etching (RIE) in a PlasmaLab μP Freon RIE tool (Plasma Technology, defunct). The size and spacing of the windows were varied to fabricate an array of cantilevers with different sizes. Finally, undercutting of the cantilevers was achieved through convex corner etching using a 20% aqueous KOH etchant at 80°C. The lateral undercut etch process is self-limiting; nevertheless, the etching was stopped once the cantilevers were fully undercut to avoid thinning the wafer. Next, we used a CHA SEC-600 e-beam evaporator (CHA Industries Inc., Fremont, CA, USA) to deposit 1.5 nm of Cr and 1.5 nm of Ni to form an adhesion layer followed by the Pd sensing layer of the desired thickness on each sample. Thicknesses were precisely controlled during deposition using a crystal monitor. The deposition rates were 1.5 and 2 Å s^−1^ for the Cr/Ni adhesion layer and the Pd sensing layer, respectively. The primary constraints on the Pd and nitride thicknesses were given by mechanical failure points and the numerical aperture of the measurement system. Very thin and long cantilevers would potentially create excellent sensors because the hydrogen-induced deflections would be large. However, in both these extremes, the cantilevers could more easily break. Additionally, to characterize accurately the structures, we need to ensure that the reflected light is captured by the objective of the DPM system. The latter effect was the primary limitation on the minimum nitride thickness and on the maximum cantilever length. A nitride thickness of 1010 nm was selected. The Pd layer was selected to be optically thick to ensure that multiple reflections did not complicate the interpretation of the DPM phase images. To study the dependence of sensor performance on Pd film thickness, two batches of devices were fabricated: one with a Pd layer thickness of 25 nm and the other with a Pd layer of 50 nm. Arrays of cantilevers were fabricated on both wafers with a variety of lengths and widths to study the dependence of sensor performance on cantilever size.

For testing, the samples were mounted in a machined aluminum test chamber. The chamber had interior dimensions of 2.5×1.7×0.64 cm, resulting in a volume of 2.7 cm^3^. The chamber had two lateral gas inlet ports located on the sides and an outlet port situated directly over the sample. The outlet port was a small hole machined through the chamber window. The hole reduces noise in the DPM measurement because it allows light to pass through without any reflection off of the glass window. Two mass flow controllers (MFCs) were used to control the hydrogen concentration and keep the chamber at a slight positive pressure. In addition, solenoid valves were also used in series with each MFC to ensure that no gas was flowing when they were set to zero flow rate. Two tubes, each having an inner diameter of 1/8 in, connected the solenoid valves to a common tube for gas mixing before splitting back to connect to the two lateral gas inlet ports of the chamber. The total tube length was 8 in, resulting in an additional volume of 1.6 cm^3^. The MFCs and solenoid valves were controlled through LabVIEW. One MFC controlled the flow rate of an already prediluted hydrogen mixture, while the other MFC set the flow rate for air, which served as the diluent. The combined flow rate was 200 sccm, which is small enough to ensure laminar flow in the chamber. Two different concentrations of diluted hydrogen were used to achieve the desired concentrations: 4% hydrogen in nitrogen (SJ Smith Co., Davenport, IA, USA; UC3344) for the [0.2%, 1%] hydrogen experiments and 0.2% hydrogen in air (Airgas Inc., Radnor Township, PA, USA; X02AI99C30053D2) for the [250 p.p.m., 0.2%] hydrogen experiments. The testing times varied, but a standard measurement consisted of exposure times of 30 min for both the test concentration and the subsequent recovery in air. All of the gas flow was automated via LabVIEW and was synced to the time stamp on the DPM images for analysis. For these measurements, a 405 nm laser (Thorlabs Inc., Newton, NJ, USA; S3FC405) was used as the illumination source.

## Results

**Figure 2:**
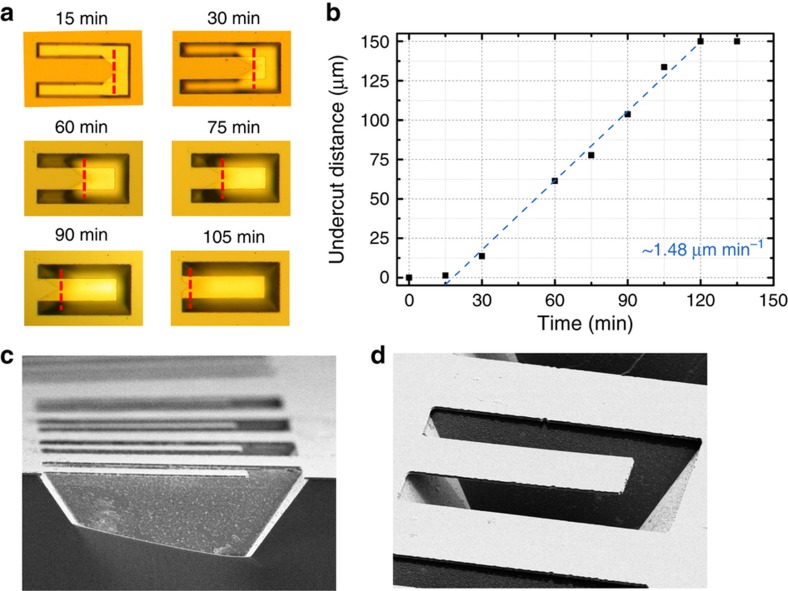
(**a**) Optical microscope images of the cantilever undercutting process at various points in time. (**b**) Undercut etch distance versus time. Scanning electron microscope (SEM) images of (**c**) cross-section view and (**d**) isometric view of a final fabricated cantilever.

Figure 2a shows the undercutting process used to fabricate the suspended structure at various points in time. Figure 2b shows the undercut etch distance versus time. Scanning electron microscope (SEM) images of one of the final structures after Pd deposition can be seen in Figures 2c and d. Although not intentional, the sloped bottom of the cavity as a result of the undercutting process is beneficial in that it prevents reflections from the cavity from interfering with the measurement in the case of partially transparent Pd films. SEM imaging also showed a slight initial upward curvature to the cantilevers that was later confirmed with DPM. Measurements of the film stress for the nitride showed that the layer was compressively strained, with a measured stress value of ~+190 MPa. This curvature was largely independent of the Pd film, implying that it is a result of a stress gradient in the nitride and is not because of relaxation kinetics of the final bimorph structure after fabrication. Because the stress in the deposited nitride film decreases as distance from the substrate increases, the top is more tensile than the bottom, resulting in upward curvature after undercutting.

**Figure 3:**
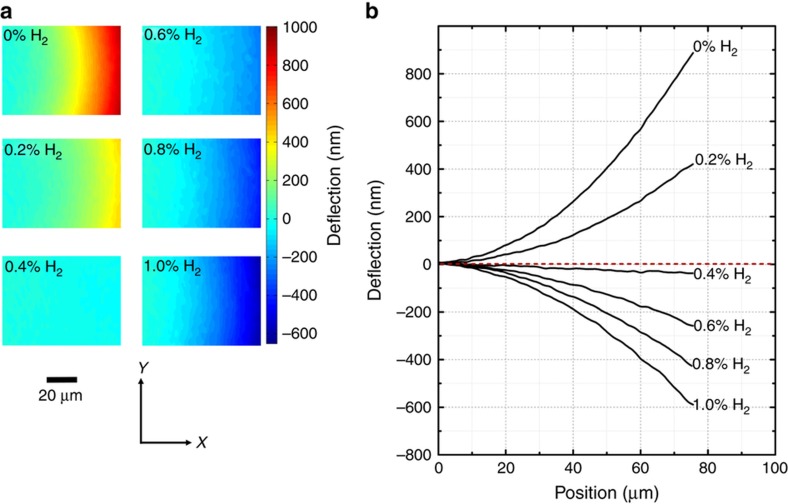
(**a**) Diffraction phase microscopy (DPM) topography at various test concentrations. Noise values were <3 nm. (**b**) Average cross-section profiles of a 40×70 μm cantilever with 50 nm of palladium (Pd) at various test concentrations. Note that the cross-section profiles of the cantilever are not to scale. The maximum deflection at the tip is only 2% of the cantilever’s length.

The topographic profile of the entire cantilever can be measured using DPM. Compared with point or bulk area measurements, such as reflected or transmitted power spectra of a cavity structure, DPM imaging allows for the calculation of quantities such as curvature, enabling more complete modeling of the cantilever. This information is extremely useful for optimizing and designing these types of sensors as well as for improving the signal-to-noise ratio in the hydrogen concentration measurements. To conduct the *in situ* measurements with hydrogen exposure, the samples were mounted in a flow chamber and exposed to a controlled mixture of hydrogen and air. The chamber was cycled with purge phases consisting of 0% hydrogen and test phases at various concentrations. Each phase lasted for 30 min, and a constant total flow rate of 200 sccm was used. Figure 3a shows the surface height maps of the 40×70 μm cantilever with 50 nm of Pd obtained with DPM during exposure to several hydrogen concentrations. Side profiles of the cantilever at these test concentrations, shown in Figure 3b, were obtained by averaging only the center (20 μm) portion of the cantilever where the curvature in the *y* direction was minimal. As expected, upon absorption of hydrogen, the Pd layer expands, resulting in a downward deflection of the cantilever. The initial curvature of 0.28 mm^−1^ is a result of the residual stress in the nitride film. This particular cantilever showed a 1.5 μm deflection at the tip and a 0.48 mm^−1^ change in curvature for 1% hydrogen. Supplementary Movie M1 shows a time lapse of the cantilever’s instantaneous height profile during hydrogen exposure.

**Figure 4:**
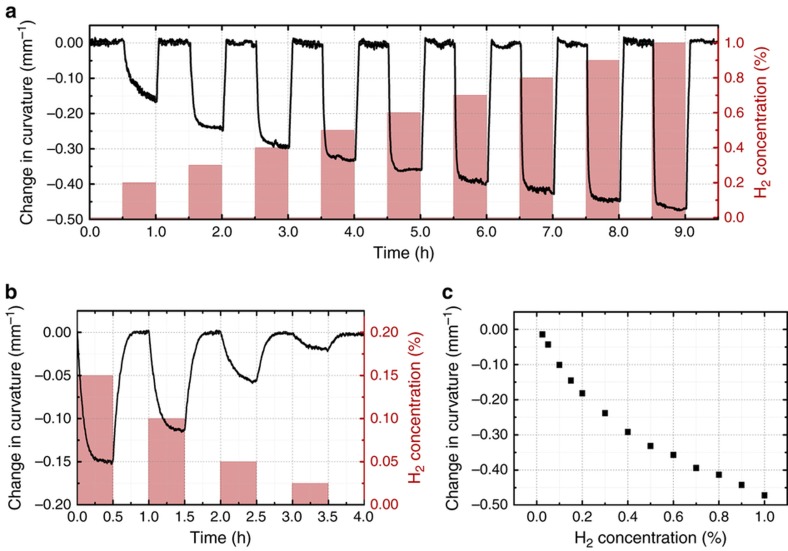
(**a** and **b**) Temporal response of change in curvature to a pulse train of H_2_ at two sets of hydrogen concentrations of [0.2%, 1.0%] and [250 p.p.m., 0.2%], respectively. Dimensions of the cantilever: 40×70 μm, *h*_Pd_=50 nm and *h*_s_=1010 nm. (**c**) The measured change in curvature versus hydrogen concentration. *h* is the layer thickness; subscripts ‘s’ and ‘Pd’ denote the SiN*_x_* substrate and the Pd film.

Based on experiments and physical models, it can be shown^45^ that the deflection of a stressed cantilever as a function of position *x* can be approximated by a quadratic

(1)z≃12Rx2+θx+z0

where *R* is the radius of curvature, *z*_0_ is the initial height, and *θ* represents the initial angle at the anchor point caused by stresses. By fitting a measured cross-section in Figure 3b to a quadratic function, the second-degree coefficient can be used to calculate the curvature *κ*=1/*R*. In addition to the topographic information, the *in situ* DPM measurement allows for the collection of temporal data. By calculating the cross-section profile at each time step and fitting to a quadratic, the curvature, as a function of time, can be extracted during hydrogen exposure. These results are shown in Figures 4a and b for two different sets of test concentrations. Figure 4c contains a summary of the curvature change. By using the curvature rather than a point deflection, data from the entire length of the cantilever are used, increasing the accuracy of the sensor. It can also be shown that the change in curvature is directly related to HILE. Hence, sensor to sensor variations, such as angle at the contact point, initial height or initial curvature, can be removed by considering the change in curvature rather than the *z*-position of a point on the cantilever. The curve in Figure 4c was inverted and used to calibrate the sensor for the subsequent experiment.

**Figure 5:**
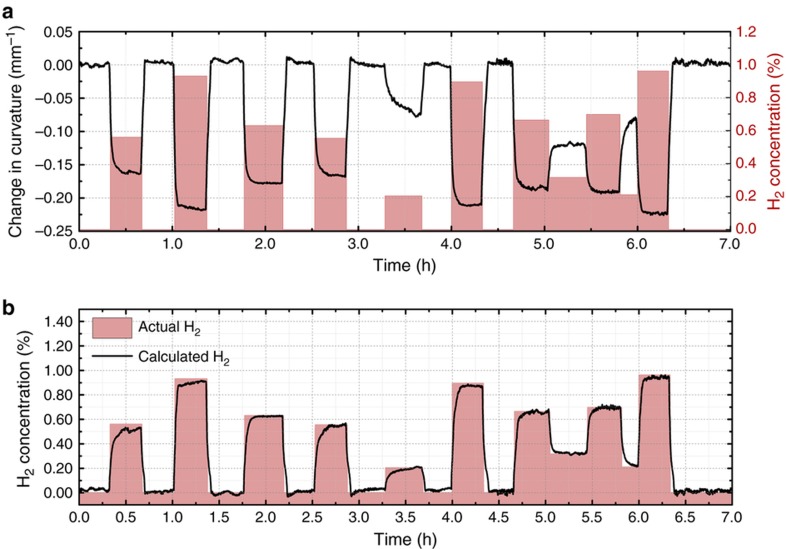
(**a**) Temporal response of the measured change in curvature for a random pulse train of H_2_. Dimensions of the cantilever: 40×70 μm, *h*_Pd_=50 nm and *h*_s_=1010 nm. (**b**) Calculated H_2_ concentration using the measured change in curvature and the calibration data shown in Figure 4c. *h* is the layer thickness; subscripts ‘s’ and ‘Pd’ denote the SiN*_x_* substrate and the Pd film.

A set of random concentrations was selected to test the predictability of the sensor for both return to zero measurements, as well as sequential random concentrations. The duration of each pulse was limited to a minimum of 20 min so that the sensor had time to fully respond. However, given that the 10–90% response times are on the order of 5 min, shorter pulses could have been used with the introduction of slightly more error. The measured curvature for the random pulse test can be seen in Figure 5a. Using the calibration, the ambient H_2_ concentration was calculated using the measured curvature. These results are shown in Figure 5b. There is a very small offset to the response at 0% that occurs when recovering from hydrogen exposure in that the cantilever returns to a state with compressive stress in the Pd layer resulting in more upward deflection. After some time, depending on the concentration, this is released and the baseline returns to normal. The error in the calculated hydrogen concentration has a value between 0 and −0.04%; that is, the determined hydrogen concentration is always less than the actual value. The error is relatively large compared with the noise in the measured height and curvature values; these factors alone correspond to an uncertainty of 0.003% H_2_ for this cantilever. Additionally, although the applied H_2_ concentrations for pulses 1, 3, 4, 7, and 8 in Figure 5b were almost the same, the pulse shapes for the recovered hydrogen concentration were different. These observations suggest systemic sources of error such as the memory effect of the Pd film that was discussed previously, which is most likely the result of the chemistry and thermodynamics of the film.

## Discussion

Here, the Pd layer is in the thin film regime, so we can use Stoney’s equation^46,47^ to calculate the change in the residual film stress, Δ*σ*_Pd_,

(2)ΔσPd(H)=Eshs26hPd(1−υs)Δκ(H)

where Δ*κ*(*H*) is the curvature change of the cantilever. *H* represents the hydrogen concentration, *E* is Young’s modulus, *h* is the layer thickness, and *υ* is Poisson’s ratio. The subscripts ‘s’ and ‘Pd’ denote the SiN*_x_* substrate and the Pd film, respectively. Here, the SiN*_x_* layer has a Young’s modulus of *E*_s_=250 GPa and a Poisson’s ratio of *υ*_s_=0.23 (Ref. 48).

**Figure 6:**
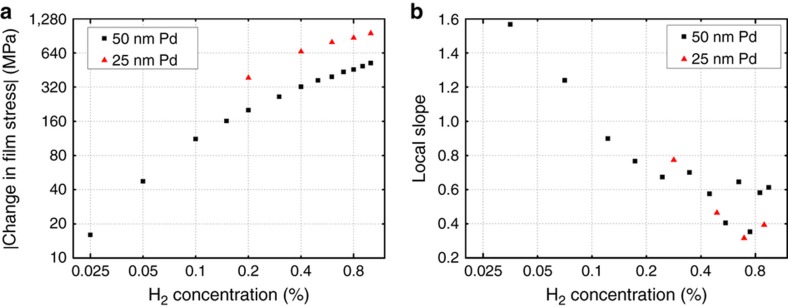
(**a**) The absolute value of the calculated change in film stress versus hydrogen concentration on a log–log scale. Dimensions of the cantilevers: 40×70 μm, *h*_s_=1010 nm, with *h*_Pd_=50 and 25 nm, respectively. (**b**) The local slope of (**a**) versus hydrogen concentration on a lin–log scale. *h* is the layer thickness; subscripts ‘s’ and ‘Pd’ denote the SiN*_x_* substrate and the Pd film.

The absolute value of the calculated change in film stress, |Δ*σ*_Pd_(*H*)|, is plotted versus hydrogen concentration on a log–log scale in Figure 6a. Data are not available for the 25 nm sample at the lower concentrations because the sample was damaged while attempting a different experiment. In the [0.2%, 1.0%] range, the change in the residual stress for the 25 nm Pd film is approximately double that for the 50 nm film. This result is to be expected if one assumes that the linear expansion coefficient is the same for the two films.

The log–log plot shown in Figure 6a is not linear with respect to hydrogen concentration over the full measurement range. To quantify the variation in slope, we define the local slope, *m*, as:

(3)m=∂ln|ΔσPd(H)|∂lnH

and use the central finite difference to approximate this derivative. The results are plotted in Figure 6b. The local slope begins much higher than 1.0 at very low concentrations and decreases to values between 0.35 and 0.65 at higher concentrations. We attribute this decrease to the transition from the pure *α* phase to the mixed *α*+*β* phase because the lattice expansion dynamics are known to be different in the two phases^31^. To some extent, the local slope is a measure of the cantilever’s sensitivity to hydrogen. Thus, we observe greater sensitivity at very low hydrogen concentrations.

## Conclusion

In conclusion, we have shown that by measuring the instantaneous height profile of a Pd thin film cantilever to nanometer-level accuracy using DPM, we can quantify small changes in curvature and thereby determine the ambient hydrogen concentration. This optomechanical hydrogen gas sensor has both a low minimum detection limit as well as the ability to measure accurately the concentrations of a random sequence of hydrogen exposures. Further, the effect of hydrogen-induced lattice expansion on the residual stress in the Pd film was also determined. Understanding the nanoscale dynamics of Pd and other thin film materials is essential in the design of future optomechanical devices and novel sensor structures. We anticipate that the results and techniques presented in this paper will stimulate further research in these areas.
