# Supplementary material for: Realization of palladium-based optomechanical cantilever hydrogen sensor
Source: Microsyst Nanoeng. 2017 Mar 27;3:16087. doi: 10.1038/micronano.2016.87 (PMC6445021; doi:10.1038/micronano.2016.87)
Supplement: Supplementary Information [file micronano201687-s1.pdf]

Supplementary file

Realization of palladium-based optomechanical cantilever hydrogen sensor

Steven J. McKeown, Xiaozhen Wang, Xin Yu and Lynford L. Goddard

Microsystems & Nanoengineering (2017) **3,** 16087; doi:10.1038/micronano.2016.87; Published online: 27 March 2017

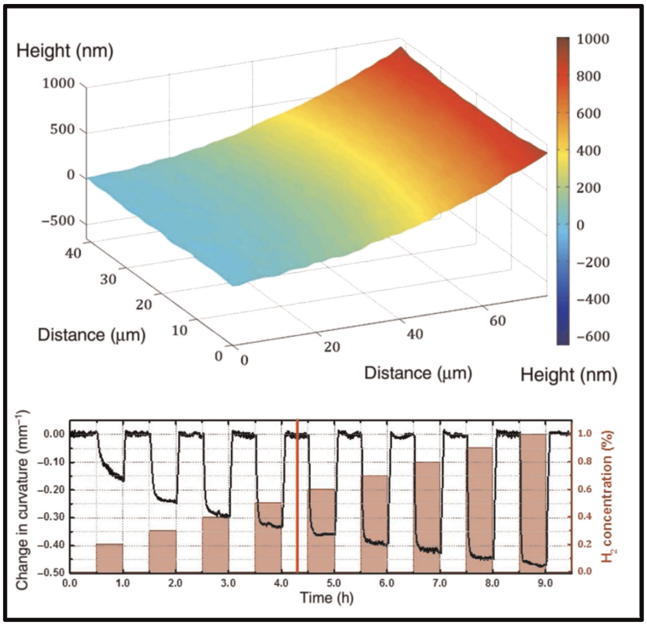

Figure S1 A still frame image from the time lapse Movie M1 of the cantilever deflection during hydrogen exposure. The top figure shows the instantaneous height profile in nanometers. The bottom figure shows the applied hydrogen concentration and the extracted curvature. Dimensions of the cantilever: 40×70 μm, $h_{Pd} = 50$ nm and $h_s = 1010$ nm. h is the layer thickness; subscripts 's' and 'Pd' denote the SiN_x substrate and the Pd film.

Photonic Systems Laboratory, Department of Electrical and Computer Engineering, Micro and Nanotechnology Lab, University of Illinois at Urbana-Champaign, 208 North Wright Street, MNTL 2254, Urbana, IL 61801, USA.
Correspondence: Lynford L. Goddard (lgoddard@illinois.edu)
